# *N*^6^-methyladenosine (m^6^A)-circHECA from secondary hair follicle of cashmere goats: identification, regulatory network and expression regulated potentially by methylation of its host gene promoter

**DOI:** 10.5713/ab.24.0081

**Published:** 2024-08-22

**Authors:** Jincheng Shen, Taiyu Hui, Man Bai, Yixing Fan, Yubo Zhu, Qi Zhang, Ruqing Xu, Jialiang Zhang, Zeying Wang, Wenxin Zheng, Wenlin Bai

**Affiliations:** 1 College of Animal Science and Veterinary Medicine, Shenyang Agricultural University, Shenyang 110866, China; 2 State Key Laboratory for Herbivorous Livestock Genetic Improvement and Germplasm Innovation of Ministry of Science and Technology and Xinjiang Uygur Autonomous Region, Urumqi 830011, China; 3 Xinjiang Academy of Animal Sciences, Urumqi 830011, China

**Keywords:** Cashmere Goats, CircHECA, *N*^6^-Methyladenosine, Promoter Methylation, Regulatory Network, Transcriptional Pattern

## Abstract

**Objective:**

The objective of this study was to identify the *N*^6^-methyladenosine (m^6^A)-circHECA molecule in secondary hair follicles (SHFs) of cashmere goats, and generate its potential regulatory network, as well as explore the potential relationship between transcriptional pattern of m^6^A-circHECA and promoter methylation of its host gene (*HECA*).

**Methods:**

The validation of circHECA m^6^A sites was performed using methylation immunoprecipitation (Me-RIP) along with reverse transcription-quantitative polymerase chain reaction (RT-qPCR) technique. The nucleus and cytoplasm localizations of m^6^A-circHECA were performed using SHF stem cells of cashmere goats with RT-qPCR analysis. Based on in-silico analysis, the regulatory networks of m^6^A-circHECA were generated with related signal pathway enrichment. The methylation level of promoter region of m^6^A-circHECA host gene (*HECA*) was assessed by the bisulfite sequencing PCR (BSP-PCR) technique.

**Results:**

The m^6^A-circHECA was confirmed to contain four m^6^A modification sites including m^6^A-213, m^6^A-297, m^6^A-780, and m^6^A-927, and it was detected mainly in cytoplasm of the SHF stem cells of cashmere goats. The integrated regulatory network analysis showed directly or indirectly complex regulatory relationships between m^6^A-circHECA of cashmere goats and its potential target molecules: miRNAs, mRNAs, and proteins. The regulatory network and pathway enrichment indicated that m^6^A-circHECA might play multiple roles in the SHF physiology process of cashmere goats through directly or indirectly interacting or regulating its potential target molecules. A higher methylation level of promoter region of *HECA* gene in SHFs of cashmere goats might cause the lower expression of m^6^A-circHECA.

**Conclusion:**

The m^6^A-circHECA might play multiple roles in SHF physiology process of cashmere goats through miRNA mediated pathways along with directly or indirectly interaction with its target proteins. The promoter methylation of m^6^A-circHECA host gene (*HECA*) most likely was implicated in its expression inhibition in SHFs of cashmere goats.

## INTRODUCTION

Cashmere goats are widely reared domestic animals in farming and pastoral areas of northern China. They are of great economic significance to local residents [1]. As is well known, the main use of cashmere goats is cashmere and meat. The cashmere has been regarded as high-grade textile raw material [2]. Cashmere is derived from the secondary hair follicles (SHFs). The SHFs undergo a periodic cycle consisting of anagen, catagen, and telogen. During anagen, the growth of cashmere fibers is regulated precisely by a series of endogenous factors through a highly coordinated regulatory network [3]. Over the past few years, the endogenous regulation of cashmere fiber growth was extensively investigated in cashmere goats at multiple levels, including functional genes [4], miRNAs [5], lncRNAs [2], circRNAs [6], and signaling pathways [7]. Overall, however, the molecular mechanism underlying the cashmere growth remains unclear in cashmere goats. Thus, it is important to identify and characterize novel regulatory molecules that may be essentially involved in cashmere fiber growth of cashmere goats.

*N*^6^-methyladenosine (m^6^A) is the most common modification in linear RNA molecules. In recent years, a considerable number of circRNAs were identified from cashmere goat SHFs [8]. Interestingly, it was revealed that extensive m^6^A modification sites also exist within some circRNA molecules in different species, and they are significantly involved in the circRNA functional exertion [9,10]. In cashmere goats, several m^6^A-circRNAs were also identified from SHFs with differential expression pattern during telogen, anagen, and catagen stages, such as m^6^A-circRNA-ZNF638, -TULP4, -DNAJB6, and -STAM2 [11,12]. This implies that m^6^A-circRNAs may play essential roles in the SHF physiological processes of cashmere goats via m^6^A-dependent mechanism. However, the m^6^A-circRNAs of cashmere goats have been less extensively investigated, and there is still a poor understanding of expression and regulatory characterizations of m^6^A-circRNAs in SHFs of cashmere goats that might be significant for revealing the molecular mechanisms of maintaining SHF cashmere growth in cashmere goats.

The circHECA was previously identified as chi_circ_2092 (HECA defined as its host gene) from the skin tissues of cashmere goats with the higher expression at anagen skin tissues compared with the counterpart of telogen [8]. In the present investigation, we revealed the molecular characterization of circHECA molecule with a validation of its potential m^6^A modification sites. Further, we generated its integrated functional regulatory network. Also, we explored the potential relationship between the expression pattern of m^6^A-circHECA and promoter methylation status of its host gene (*HECA*) during SHF cycles of cashmere goats. Our results would provide novel information for elucidating the biological significance and functional regulatory characteristics of m^6^A-circHECA in SHF regeneration with the morphogenesis and growth of cashmere fibers in cashmere goats.

## MATERIALS AND METHODS

### Preparation of samples and extraction of total RNA and genomic DNA

The experimental procedures have been reviewed and approved by the Experimental Animal Ethics and Welfare Committee of Shenyang Agricultural University with the ethical code: 2023030208. Also, all experiments were carried out following the approved protocol guidelines. Nine clinically healthy cashmere goats (adult, female, and no traceable genetic relationships) were selected from the breeding center of Liaoning cashmere goat (Liaoyang, Liaoning, China). Using sterile scalpel blades, we collected skin tissues from the body side part of the selected goats at three stages of SHF cycles: anagen, catagen, and telogen as described in our previous study [12]. The skin tissue sample from each goat was subjected to clearing with alcohol of 75%. Subsequently, the skin tissue was cut into pieces (5 mm^2^) with phosphate-buffered saline washing three times. The samples were further subjected to digesting with dispase II of 0.25% (Roche, Mannheim, Germany) at 4°C overnight. Ultimately, the SHFs were isolated from digested skin tissue using disposable syringe needle and sterilized scalpel under a stereo-microscope (Leica, Wetzlar, Germany). The total RNA was isolated from SHFs of each sample using the RNAiso reagent kit (TaKaRa, Dalian, China), and the genomic DNA were isolated using the TIANamp Genomic DNA Kit (TIANGEN, Beijing, China).

### Transcription source of m^6^A-circHECA with its molecular characteristics analysis

The circHECA has been identified from skin tissues of cashmere goats in a previous investigation [8]. The BioEdit software (Version 7.0.5) [13] was used for the managing and displaying of circHECA sequence. The transcription source of circHECA was defined based on an aligning of its linear sequence against the goat genome datasets (Genome assembly ARS1.2, https://www.ncbi.nlm.nih.gov/datasets/genome/GCF_001704415.2, last access: 08 November 2023). We predicated the potential m^6^A sites on circHECA sequence with the SRAMP online program ( https://www.cuilab.cn/sramp, last access: 09 November 2023). The potential binding miRNAs within circHECA sequence were predicted using the custom sub-procedure of miRDB online service program ( https://www.mirdb.org, last access: 09 November 2023), along with the online service database: miRNAsong ( https://www2.med.muni.cz/histology/miRNAsong/index.php, last access: 09 November 2023). In the prediction analysis, here, due to the unavailability of goat data in miRDB database, alternatively, we used the human data for performing the prediction with removing the potential target miRNAs unidentified in goats. The coding potential of circHECA sequence was analyzed using two programs: Coding Potential Assessment Tool (CPAT, https://lilab.research.bcm.edu/cpat/index.php, last access: 09 November 2023) and Coding Potential Calculator (CPC, https://cpc.cbi.pku.edu.cn, last access: 09 November 2023). The open reading frame (ORF) of circHECA sequence was analyzed using ORF Finder program at NCBI website ( https://www.ncbi.nlm.nih.gov/orffinder, last access: 09 November 2023) along with the BioEdit software (Version 7.0.5) [13].

### The m^6^A site validation of circHECA and its subcellular localization analysis in SHF stem cells

The m^6^A site validation of circHECA was performed using methylation immunoprecipitation (Me-RIP) along with reverse transcription-quantitative polymerase chain reaction (RT-qPCR) technique. We carried out the Me-RIP analysis of m^6^A-circHECA following the described methods in previous publication [14]. In short, the total RNA sample (100 μg) was incubated with RNase R (Geneseed, Guangzhou, China) at 37°C for 3 min, and was further subjected to concentration using the Monarch RNA Cleanup Kit (NEB, Ipswich, MA, USA). And then, the obtained RNA sample was fragmented by the NEBNext Magnesium RNA Fragmentation Module (NEB, USA), followed by a concentration using the Monarch RNA Cleanup Kit (NEB, USA). The fragmented RNA sample of 2 μg was used as input control. At 4°C, half fragmented RNA product was incubated with anti-m^6^A antibody (2 μg; Synaptic Systems, Gottingen, Germany) or immunoglobulin G (2 μg; Cell Signaling Technology, Danvers, MA, USA) for 4 h. Subsequently, The Dynabeads Protein A (Thermo Scientific, Rockford, IL, USA) was incubated with the RNA-antibody complex at 4°C for 2 h. Finally, the RNA was isolated with the RNAiso reagent kit (TaKaRa, China). Using random primers, the first strand cDNAs were synthesized by the M MuLV cDNA synthesis kit (Sangon, Shanghai, China). Based on the site-specific primers (Table 1), the enrichment abundance of each m^6^A site within circHECA molecule was measured by RT-qPCR technique where the relative enrichment of each m^6^A site of circHECA was normalized to the input control [14].

The nucleus and cytoplasm localizations of m^6^A-circHECA were performed using SHF stem cells of cashmere goats stored in our laboratory as described in our previous publication [1]. The cells were cultured in Dulbecco’s modified Eagle medium (DMEM)/F12 medium with 10% fetal bovine serum (Hyclone, Logan, UT, USA). The incubator was set as 37°C with the concentration of CO_2_ being 5%. The medium was changed every 2 days. The cytoplasmic and nuclear RNA was isolated from SHF stem cells by Cytoplasmic and Nuclear RNA Purification Kits (AmyJet, Wuhan, China). Subsequently, the relative expression of circHECA, GAPDH and snRNA-U6 was measured in cytoplasm and nucleus of SHF stem cells using RT-qPCR technique. Here, the expression level of GAPDH and snRNA-U6 was measured to serve as the cytoplasm and nuclear control, respectively. In RT-qPCR analysis, the relative expression of the analyzed genes was calculated with the 2^−ΔΔCt^ method.

### The regulatory network analysis of m^6^A-circHECA with related signal pathway enrichment

As described above, six potential target miRNAs of m^6^A-circHECA had been predicted using the custom sub-procedure of miRDB along with the miRNAsong procedure including chi-miR-20b, chi-miR-27a-5p, chi-miR-129-3p, chi-miR-187, chi-miR-449a-5p, and chi-miR-449b-3p. For the competitive endogenous RNA (ceRNA) regulatory network of m^6^A-circHECA, the potential target genes of the six miRNAs (chi-miR-20b, chi-miR-27a-5p, chi-miR-129-3p, chi-miR-187, chi-miR-449a-5p, and chi-miR-449b-3p) were further predicted using the custom prediction sub-procedure of miRDB along with the miRNAsong procedure as recommended in previous publication [11]. Finally, the ceRNA regulatory network of m^6^A-circHECA was generated and visualized using the Cyotoscape (Version 2.8.3) procedure [15]. The CluePedia built-in plugin in Cyotoscape procedure was used for enriching the m^6^A-circHECA regulatory genes mediated by the six predicted miRNAs into signaling pathways with the default settings ( https://www.ici.upmc.fr/cluepedia/, last access: 20 November 2023). CluePedia generated a regulatory network connecting the analyzed genes and implicated signaling pathways which indicated novel potential regulatory relationships among the analyzed genes and involved pathways [16].

For the interactional network of m^6^A-circHECA with potential target proteins, we performed a prediction within m^6^A-circHECA sequence to screen the potential interactional proteins using RNA-protein Interaction Prediction (RPISeq). The RPISeq ( https://pridb.gdcb.iastate.edu/RPISeq/batch-rna.html, last access: 24 November 2023) can computationally screen potential interactional proteins within given RNA sequence based on sequence-derived pattern [17]. The obtained interactional relationships of m^6^A-circHECA with potential target proteins were presented as a network that was further deeply explored using an online service procedure: FunRich ( www.funrich.org, last access: 24 November 2023). The potential target proteins directly or indirectly regulated by m^6^A-circHECA were further enriched into signaling pathways using the CluePedia procedure with default settings [16]. Ultimately, the significantly enriched pathways were presented as a chordmap that was generated by an online platform for data analysis and visualization ( https://www.bioinformatics.com.cn, last access: 24 Nov 2023) [18].

### Expression analysis of m^6^A-circHECA and methylation detection of its host gene promoter in SHF cycles of cashmere goat

Using the M-MuLV cDNA synthesis kit with random primers (Sangon, Shanghai, China), the reverse-transcriptions were performed on total RNA extracted from cashmere goat SHFs during different stages (telogen, anagen and catagen). For an expression test of the m^6^A-circHECA in SHFs of cashmere goats, we performed the RT-qPCR analysis with the divergent primers (Table 1) in a light Cycler 480 real-time PCR system (Roche Diagnostics, Germany). In a final volume of 25 μL, the qPCR reactions were performed where the reaction system were comprised of Green Premix Ex Taq II of 12.5 μL TB (Tli RNaseH Plus; TaKaRa, Chnia), the first-strand cDNA solution of 2.0 μL, each primer of 1.0 μL (10 μ*M*), and ddH_2_O water of 8.5 μL. In qPCR reactions, the thermal cycling parameters were set as a single cycle of 95°C for 3 min, followed by 40 cycles (95°C for 5 s, 57°C for 30 s, and 72°C for 30 s). The relative express of m^6^A-circHECA was normalized to the GAPDH and calculated with the 2^−ΔΔCt^ method [19].

For assessing the methylation level of promoter region of m^6^A-circHECA host gene (*HECA*), we hunted potential a CpG island within the 800-bp region immediately upstream of transcription start sites (TSS) in *HECA* gene using Methyl Primer Express program (Version 1.0; Applied Biosystems, Foster City, CA, USA). We analyzed the potential binding sites of transcription factors using JASPAR program ( https://jaspar.elixir.no, last access: 25 Nov 2023). The extracted genomic DNA from each goat was treated by the MethylCode Bisulfite Conversion Kit (Invitrogen, Shanghai, China), and pooled into three groups: telogen, anagen and catagen of SHF cycle of cashmere goats. Subsequently, under the described above assay, bisulfite sequencing PCR (BSP-PCR) reactions were performed with BSP-primers (Table 1). After purifying with DNA purification kit (TaKaRa, China), the obtained BSP-PCR products were ligated to pMD18-T Vector (TaKaRa, China), followed by a propagation in competent *Escherichia coli* DH5α cells. For each analyzed stage (telogen, anagen, and catagen) of SHF cycle of cashmere goats, we sequenced ten positive clones. The resultant results were displayed by the QUMA procedure [20].

### Statistical analysis

The SPSS 17.0 software (SPSS Inc., Chicago, IL, USA) was used to conduct the statistical analysis for the obtained data. We presented the analyzed results using the GraphPad Prism version 8.3.0 for Windows, GraphPad Software, San Diego, CA, USA ( www.graphpad.com, last access: November 27, 2023). The difference between analyzed groups was compared with Student’s *t*-test where p-value less than 0.05 was considered as significant difference statistically.

## RESULTS AND DISCUSSION

### Transcription source of m^6^A-circHECA with its sequence analysis in cashmere goats

**Figure 1:**
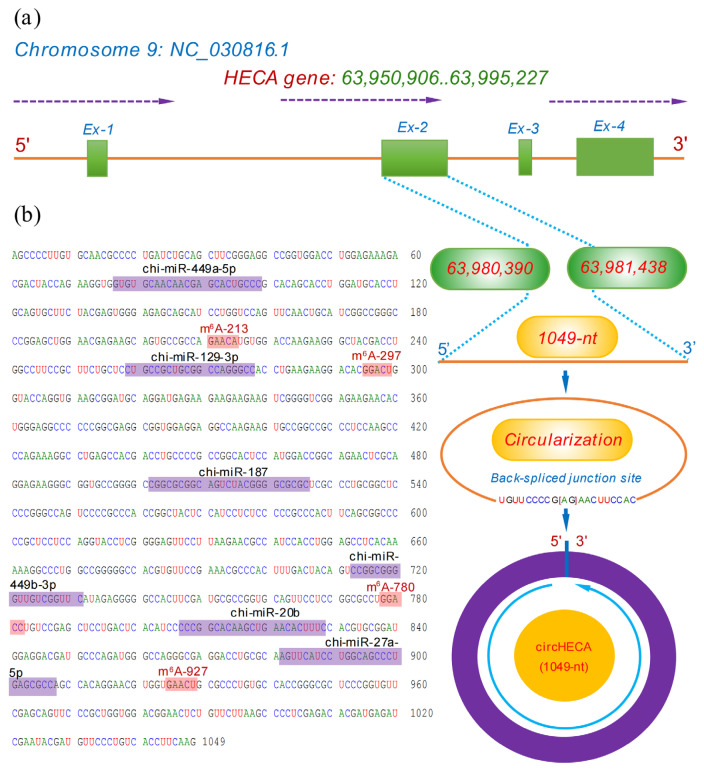
Host source of circHECA in cashmere goats with its sequence structural features. (a) Structural diagram of the host gene of circHECA with its reverse splicing size of 1,619-nt in length. (b) Display of circHECA sequence that contains the potential binding sites of six miRNAs including chi-miR-449a-5p, chi-miR-129-3p, chi-miR-187, chi-miR-449b-3p, chi-miR-20b, and chi-miR-27a-5p. Also, four potential m^6^A modification sites were harbored within m^6^A-circHECA sequence including m^6^A-213, m^6^A-297, m^6^A-780, and m^6^A-927 with the motif of GAACA, GGACU, GGACC, and GAACU, respectively (For interpretation of the references to colour in this figure legend, the reader is referred to the web version of this article.).

The circHECA (also known as chi_circ_2092) was previously identified from skin tissue of cashmere goat with a spliced length of 1,049-nt [8]. To define the transcription source of m^6^A-circHECA, we performed an alignment of its linear sequence against the goat genome datasets. As a result, the *HECA* on chromosome 9 was revealed to be its host gene (Figure 1a). As annotated well in National Center for Biotechnology Information (NCBI, https://www.ncbi.nlm.nih.gov ), the goat *HECA* gene is composed of four exons, including exons 1, 2, 3 and 4. Whereas, m^6^A-circHECA is formed by reverse splicing of its entire exon 2 with position nos. 63,980,390–63,981,438 of the NC_030816.1 sequence (Figure 1a).

Based on in-silico analysis, four potential m^6^A modification sites were revealed in m^6^A-circHECA including m^6^A-213, m^6^A-297, m^6^A-780, and m^6^A-927 with the motif of GAACA, GGACU, GGACC, and GAACU, respectively (Figure 1b). These motif structures of m^6^A-circHECA are fully in line with the m^6^A motif: RRACH (R: A/G and H: A/C/U) previously found in linear RNAs [21]. On the other hand, bioinformatively, we also found that m^6^A-circHECA harbored potential binding target sites of several miRNAs, including chi-miR-449a-5p, chi-miR-129-3p, chi-miR-187, chi-miR-449b-3p, chi-miR-20b, and chi-miR-27a-5p (Figure 1b), which implies that these miRNAs may be implicated in the functional performance of m^6^A-circHECA in SHF physiological process of cashmere goats as shown in previous investigation [1,12].

### The m^6^A site validation and subcellular localization of circHECA in SHF stem cells of cashmere goats along with *in-silico* analysis on binding features with its potential target miRNAs

**Figure 2:**
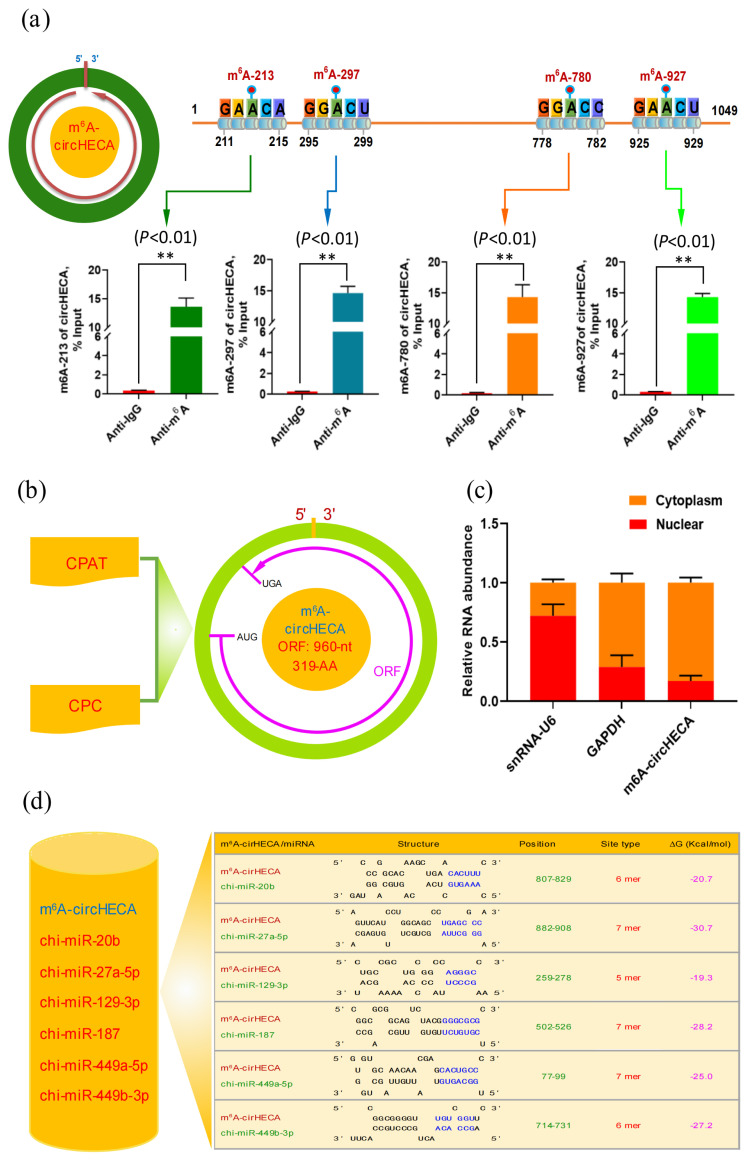
The m^6^A site validation and subcellular localization of circHECA in SHF stem cells of cashmere goats along with in-silico analysis on binding features with its potential target miRNAs. (a) The distribution diagram of m^6^A sites within circHECA sequence and their validation. (b) The coding potential analysis of m^6^A-circHECA molecules using two programs CPAT and CPC. The open reading frame (ORF) of circHECA sequence was analyzed using ORF Finder program at NCBI website ( https://www.ncbi.nlm.nih.gov/orffinder) along with the BioEdit program (Version 7.0.5; Hall [13]). (c) The subcellular localization of m^6^A-circHECA in SHF stem cells of cashmere goats. The GAPDH and snRNA-U6 was used as the cytoplasm and nuclear control, respectively. (d) The interacting miRNA analysis of m^6^A-circHECA with the potential binding sites within m^6^A-circHECA sequence (For interpretation of the references to colour in this figure legend, the reader is referred to the web version of this article.). ** p<0.01.

Based on the above predicted m^6^A sites within circHECA sequence (Figure 1b; Figure 2a), we further performed a validation for each potential m^6^A modification site by Me-RIP along with qPCR technique (Me-RIP-qPCR). As shown in Figure 2a, the four potential m^6^A modification sites were confirmed in circHECA molecule including m^6^A-213, m^6^A-297, m^6^A-780, and m^6^A-927 (Figure 2a). Interestingly, it is thought that the m^6^A modifications sites within m^6^A-circRNA molecules are essential to their functional performance in biological cells [9,22]. Thus, it can be suggested that the verified m^6^A modifications of circHECA may mean further biological significance in regulating its functional roles SHFs of cashmere goats as reported in a recent publication by Yin et al [12]. On the other hand, as well known, circRNAs were originally regarded as non-coding RNAs that had no ability of encoding protein/peptide, however, increasing lines of evidence showed that some of circRNAs have capability of encoding a protein/peptide with biological significance [23]. Based on bioinformatics analysis, therefore, we assessed the encoding potential of m^6^A-circHECA by both CPAT and CPC programs with a screening of potential ORF within its sequence. As shown in Figure 2b, m^6^A-circHECA harbored one ORF of 960-nt with encoding a potential novel HECA-319aa protein (Figure 2b). Also, there is evidence that the m^6^A motifs are heavily implicated in driving an effective translation initiation of m^6^A modification circRNAs [9]. Taken together with our results, we strongly suggest that the potential novel HECA-319aa protein should be further identified with its functional validation, which might mean further biological significance in SHF physiology of cashmere goats.

In previous investigations, it has been demonstrated that the subcellular localization of a given circRNA can provide significant clues on its functional roles in biological cells [24]. In this study, we also evaluated the subcellular localization of m^6^A-circHECA in SHF stem cell of cashmere goats. As shown in Figure 2c, although the expression of m^6^A-circHECA was detected in both nucleus and cytoplasm of the analyzed SHF stem cells, it was detected mainly in cytoplasm of the analyzed cells (Figure 2c). On the whole, it is widely accepted that the majority of circRNAs located in cytoplasm regulate the availability of miRNAs binding to target mRNA molecules [24]. Based on in-silico analysis, therefore, we further investigated the binding features of m^6^A-circHECA with the above predicted six potential target miRNAs (chi-miR-449a-5p, chi-miR-129-3p, chi-miR-187, chi-miR-449b-3p, chi-miR-20b, and chi-miR-27a-5p), including binding structure, site types, and free energy (ΔG). As shown in Figure 2d, a good sequence matching structure can be formed between m^6^A-circHECA and its target miRNA with binding site type of 5 to 7 mer and free energy (ΔG) of −30.7 to −19.3 kcal/mol (Figure 2d). Thus, we speculate that the m^6^A-circHECA may exert its functional roles via miRNA mediated pathways in SHFs of cashmere goats.

### The ceRNA network analysis of m^6^A-circHECA with signaling pathway enrichment of its potential regulatory genes

**Figure 3:**
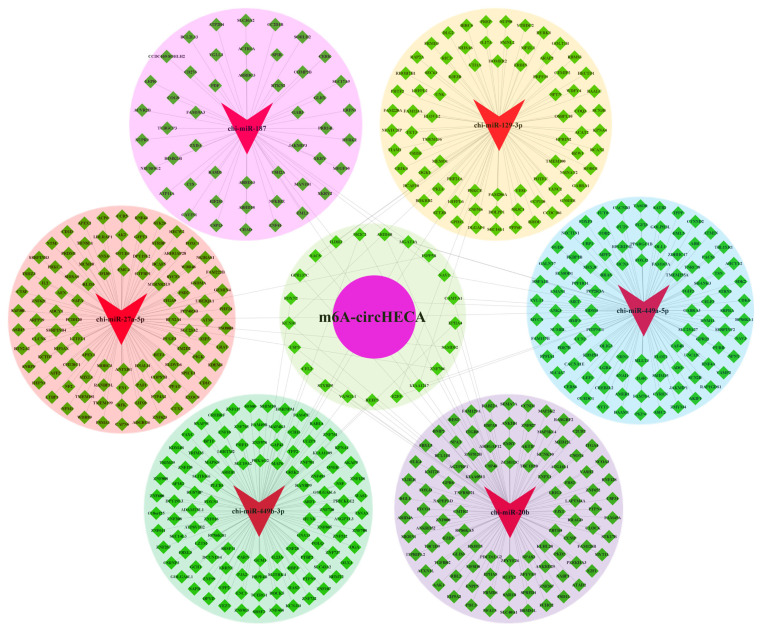
CeRNA network of m^6^A-circHECA of cashmere goat with its potential target miRNAs and their potential interacting mRNAs. The m^6^A-circHECA is indicated by a purple circle. The miRNAs were indicated by red swallowtail shapes. The potential target mRNAs of the miRNAs are indicated by green diamond. The ceRNA regulatory network was generated and visualized using Cytoscape procedure (version 2.8.3; Smoot et al [15]) (For interpretation of the references to colour in this figure legend, the reader is referred to the web version of this article.).

It is well known that circRNAs can sequester target miRNAs to inhibit the binding of the miRNAs with corresponding target mRNAs thereby ultimately regulating the expression of protein-coding genes via a ceRNA network mechanism [10]. To explore the potential functional mechanisms of m^6^A-circHECA in SHFs of cashmere goats, here, we generated a ceRNA network of m^6^A-circHECA with potentially interactive miRNAs and their corresponding target genes based on the use of bioinformatics tools. As presented in Figure 3, six miRNAs were revealed to have potential interactive relationships with m^6^A-circHECA including chi-miR-449a-5p, chi-miR-129-3p, chi-miR-187, chi-miR-449b-3p, chi-miR-20b, and chi-miR-27a-5p. Whereas, these miRNAs may be further implicated in potentially regulating the expression of corresponding protein-coding genes (Figure 3). Interestingly, the chi-miR-449a-5p, chi-miR-129-3p, chi-miR-187, chi-miR-20b, and chi-miR-27a-5p were previously identified in hair follicles of cashmere goats [25]. Moreover, the chi-miR-129-3p and chi-miR-20b were recorded to be significantly upregulated at telogen hair follicles of cashmere goats compared with the counterpart of anagen with a p-value of 0.00066 and 0.00026, respectively [25]. As well known, the telogen is a relative quiescent stage of SHFs in cashmere goats with low proliferation rate and small volume [26]. Thus, it can be suggested that the chi-miR-129-3p and chi-miR-20b may play significant roles in establishing the optimal expression balance of protein-coding genes at telogen SHFs of cashmere goats, which may be essentially important for cashmere goat SHFs to maintain the quiescent status at telogen stage. Whereas this biological process may be ultimately regulated by m^6^A-circHECA via the miRNAs (chi-miR-129-3p and chi-miR-20b) meditated pathways.

On the other hand, some of the protein-coding genes in the generated ceRNA network have been verified to play essential roles in physiological process of hair follicle. As an example, the LEF1 was found to direct the fate of hair follicle stem cells through promoting the β-catenin translocation [27]. Also, it was reported that the inhibition of CDK6 along with CDK4 in skin tissue by a membrane-transducible TAT-p16INK4a protein impeded the growth and differentiation of hair follicle [28]. Here, both LEF1 and CDK6 were revealed to be potential target genes of chi-miR-449a-5p that might interact with m^6^A-circHECA of cashmere goat (Figure 3). Similarly, the *FOXC1*, *AAK1*, and *ZNF704* were predicted as potential target genes of chi-miR-27a-5p, chi-miR-20b, and chi-miR-449b-3p, respectively. Previously, it was demonstrated that FOXC1 could not only maintain the niche of hair follicle stem cells, but also reinforce their quiescence to preserve the long-term potential of hair follicle regeneration [29]. Whereas the AAK1 and ZNF704 were also implicated in the development and growth of hair follicle [22]. Taken together with our results, it can be inferred that m^6^A-circHECA might play multiple roles in maintaining the normal SHF physiology of cashmere goat through precisely regulating the expression of its miRNA mediated target genes.

**Figure 4:**
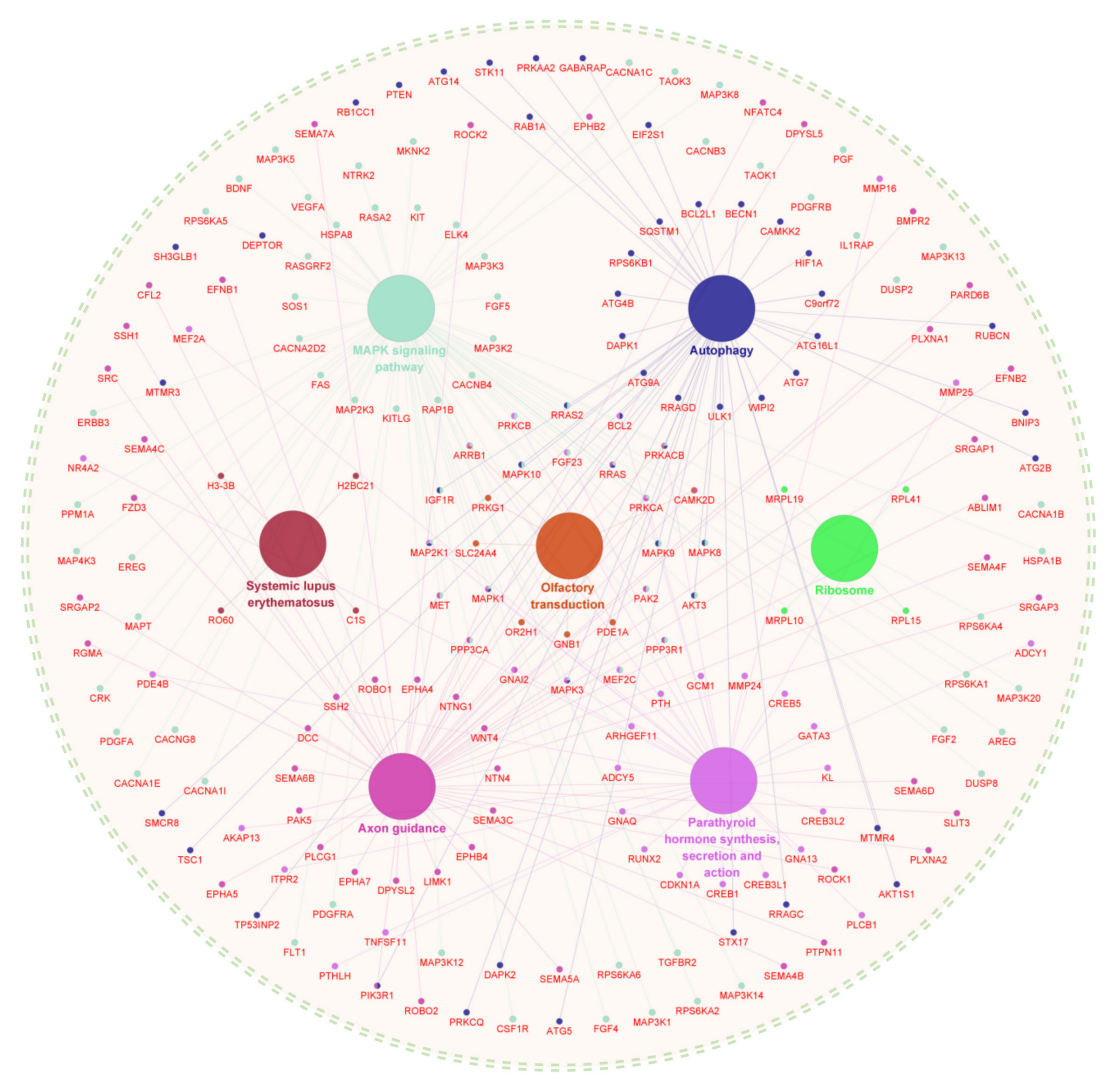
Signaling pathway enrichment of m^6^A-circHECA regulatory genes via miRNA mediated mechanism. The enrichment analysis was conducted using the CluePedia plugin in Cytoscape procedure. The generated network for enrichment pathway exhibited both the significantly enriched signaling pathways and their corresponding genes with each pathway term and associated genes sharing the same color (For interpretation of the references to colour in this figure legend, the reader is referred to the web version of this article.).

To gain a further insight into the potential functional mechanisms of m^6^A-circHECA, we performed a pathway enrichment upon its miRNA mediated target genes using the CluePedia procedure. As observed from Figure 4, the potential miRNA mediated target genes of m^6^A-circHECA were significantly enriched into multiple signaling pathways, including MAPK signaling pathway, autophagy, systemic lupus erythematosus, olfactory transduction, ribosome, Axon guidance, and Parathyroid hormone synthesis, secretion, and action (Figure 4). Of them, interestingly, several signaling pathways were previously verified to play important roles in the development and growth of hair follicles. For example, it was demonstrated that the activation of MAPK signaling pathway was heavily implicated in hair regeneration and growth with heightening expression of involved growth factors [30,31]. Also, there is evidence that the activation of axon guidance signal is required for the morphogenesis of hair follicles where it exerts function through driving the rearrangement of localized cells [32]. More recently, it was reported that the activation of autophagy pathways was also involved in promoting the hair growth of 5-Bromo-3,4-dihydroxybenzaldehyde along with activating Wnt/β-catenin pathway and inhibiting of the transforming growth factor-β (TGF-β) pathway in dermal papilla cells [33]. Thus, we inferred that the miRNA mediated regulatory genes of m^6^A-circHECA might be involved in the SHF physiological processes of cashmere goat, where their biological function might be ultimately subjected to the regulation of the m^6^A-circHECA molecules.

### Interactional regulatory network of m^6^A-circHECA of cashmere goat with its potential target proteins along with signaling pathway enrichment

**Figure 5:**
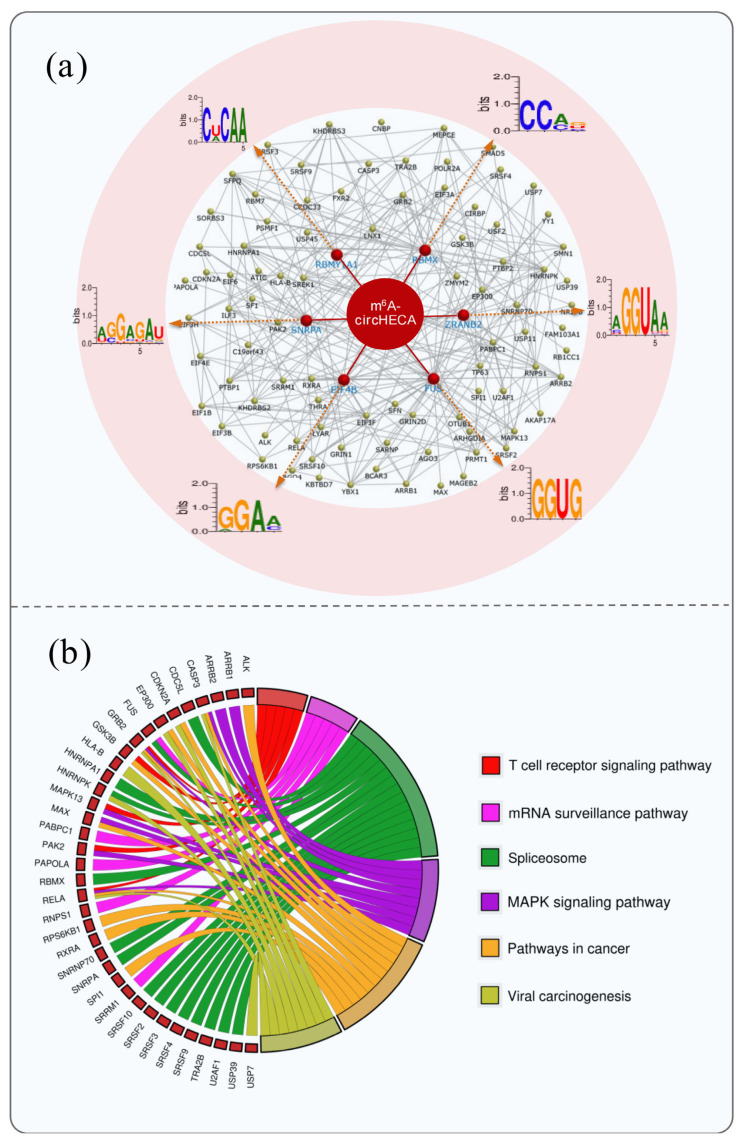
Interactional network of m^6^A-circHECA of cashmere goat with its directly and indirectly regulated target proteins along with signaling pathway enrichment. (a) Interactional regulatory network of m^6^A-circHECA of cashmere goat with its potential target proteins. The potential target proteins directly interacting with m^6^A-circHECA are indicated by bigger round balls with binding motifs with each protein being presented in the outer ring. The remaining target proteins that may be indirectly regulated by m^6^A-circHECA were indicated with smaller round balls. (b) Pathway enrichment analysis of the potential target proteins directly and indirectly regulated by m^6^A-circHECA. The enrichment for signaling pathway was carried out using the CluePedia Cytoscape plugin with default settings. the significantly enriched pathways were presented as a chordmap that was generated by an online platform for data analysis and visualization ( https://www.bioinformatics.com.cn, last access: 24 Nov 2023, Tang et al [18]) (For interpretation of the references to colour in this figure legend, the reader is referred to the web version of this article.).

Over past few years, increasing evidence indicated that the interactions of non-coding RNAs and RNA binding proteins are widely implicated in a variety of cellular processes, such as, transcription, post-transcriptional regulation of gene expression and host defense against pathogens [34]. Accordingly, to further explore the potential interactional proteins of m^6^A-circHECA in SHFs of cashmere goat, we generated an interactional regulatory network of m^6^A-circHECA of cashmere goat with its potential RNA-binding proteins. As a result, there are six proteins having a direct binding relationship with m^6^A-circHECA of cashmere goats, including RBMY1A1, RBMX, SNRPA, ZRANB2, EIF4B, and FUS (Figure 5a). As shown in Figure 5a, moreover, each of them has been revealed to have multiple further regulatory relationships with other proteins. Although, it is not yet known whether these six proteins play directly functional roles in SHFs of cashmere goats, several of their proteins were implicated in hair follicle-related key signaling pathways, such as RBMX in Wnt/β-catenin signaling pathway [35], EIF4B in MAPK signaling pathway [36] and FUS in TGF-β signaling pathway [37]. Additionally, the SNRPA is previously known as an important paralog of SNRPB2 because of its structure closely related with SNRPB2 [38]. Although it is not yet known whether SNRPA plays similar functional roles with SNRPB2 in SHFs of cashmere goats, it was demonstrated that SNRPB2 was involved in Wnt signaling pathway [39]. Whereas it is well known that the Wnt signal play an important role in the growth and development of hair follicles [40].

Also, we performed a signaling pathway enrichment on the potential target proteins directly or indirectly regulated by m^6^A-circHECA molecules. As a result, we found that the analyzed regulatory proteins of m^6^A-circHECA were significantly enriched into multiple signaling pathways including T cell receptor signaling pathway, mRNA surveillance pathway, Spliceosome, MAPK signaling pathway, pathways in cancer, and viral carcinogenesis (Figure 4b). Of them, the T cell receptor signaling pathway was implicated in T cell-mediated developmental defect of hair follicles [41]. Also, the MAPK signaling pathway was demonstrated to play important roles in regeneration and growth of hair follicles [30,31]. Taken together, we speculate that the significantly enriched proteins may be essentially implicated in the SHF growth and development of cashmere goats which may be directly or indirectly regulated by m^6^A-circHECA molecules.

### Expression pattern of m^6^A-circHECA and its relationships with methylation status of the host gene (*HECA*) promoter in SHFs of cashmere goats

**Figure 6:**
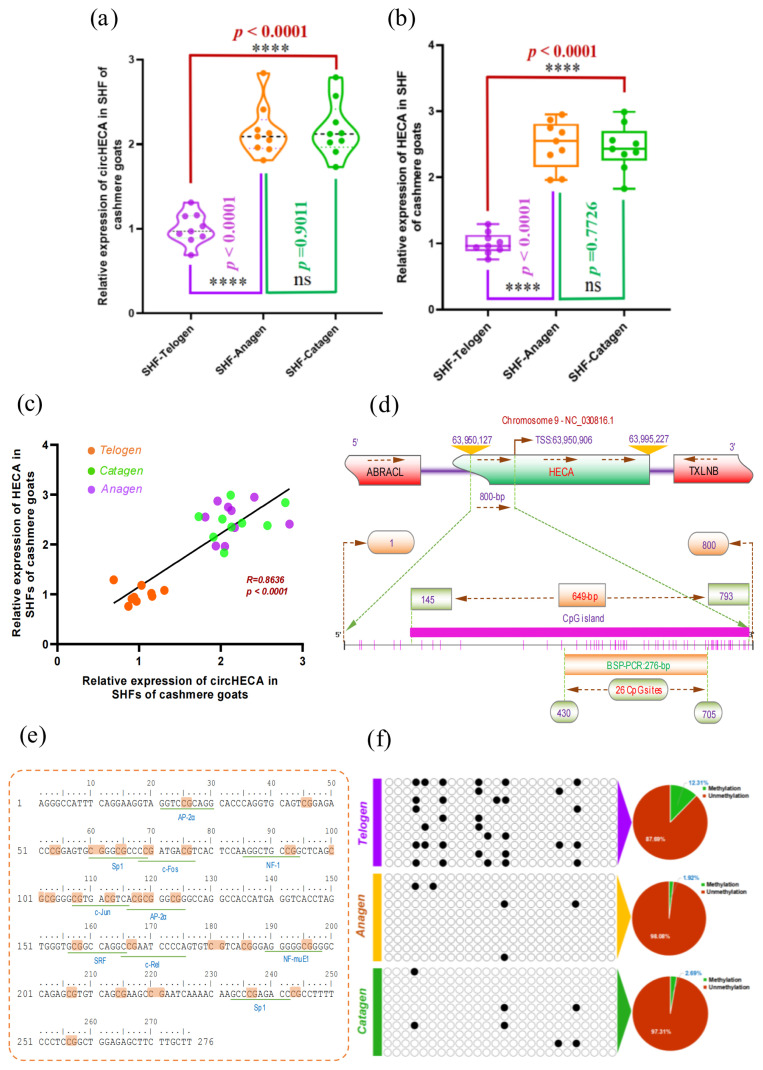
Expression pattern of m^6^A-circHECA and its relationships with methylation status of the host gene (*HECA*) promoter in SHFs of cashmer goats. (a) Relative expression of m^6^A-circHECA in SHFs of cashmere goat at telogen, anagen and catagen. (b) Relative expression of HECA genes in SHFs of cashmere goat at telogen, anagen and catagen. (c) Expression correlation of m^6^A-circHECA and its host gene *HECA* in SHFs of cashmere goats at telogen, anagen and catagen. (d) The analysis of CpG islands within *HECA* gene promoter where the CpG sites are indicated by short pink vertical lines. The nucleotide positions are indicated based on *HECA* gene sequence of goat genome datasets (Genome assembly ARS1.2, https://www.ncbi.nlm.nih.gov/datasets/genome/GCF_001704415.2). BSP represents bisulfite sequencing polymerase chain reaction. (e) The potential binding sites (underlined with green) of transcription factors in BSP amplification region of *HECA* gene promoter of cashmere goats where the CpG sites areindicated with yellow shadow regions. (f) The BSP analysis results of promoter region of *HECA* gene in SHFs of cashere goat at telogen, anagen and telogen. The methylated and unmethylated CpG sites are indicated by the black (filled) and white (unfilled) circles, respectively. For each analyzed stage of cashmere goat SHFs, the percentage of methylated CpG sites of *HECA* gene promoter are indicated with pie charts. SHFs, secondary hair follicles; BSP, bisulfite sequencing PCR. (For interpretation of the references to colour in this figure legend, the reader is referred to the web version of this article.).

It is generally thought that the biogenesis of circRNAs is highly spatio-temporal specific across different cell types and developmental stages of tissues and organs, and their generation in cells is precisely controlled at multiple layers [42]. In previous investigation, it was recorded that the expression of m^6^A-circHECA along with its host gene *HECA* in SHFs of cashmere goats was significantly higher at both anagen and catagen than the counterpart of telogen [8]. Here, this was further validated in SHFs of nine individuals from Liaoning cashmere goats (Figure 6a; 6b). In addition, as shown in Figure 6a and b, m^6^A-circHECA and its host gene *HECA* linear mRNA exhibited a highly similar expression pattern in SHFs of cashmere goat during SHF cycles: telogen, anagen and catagen. Moreover, we found that a significantly positive correlation relationship existed in expression pattern between m^6^A-circHECA and its host gene *HECA* linear mRNA molecules (Figure 6c). Although the precise biological functions of HECA linear mRNAs in SHF physiology need to be further clarified in cashmere goats, it is thought that HECA is deeply implicated in the main aspects of cellular behavior (such as growth and differentiation) through Wnt/β-catenin signaling pathway [8], while the Wnt/β-catenin signaling pathway plays key role in the growth and development of hair follicle [11]. Thus, we inferred that the HECA along with its m^6^A-circHECA might form a novel regulatory layer in the SHF physiological process of cashmere goats.

It is well recognized that the promoter methylation of protein-coding gene is essentially implicated in its expression regulation without the alteration in promoter DNA sequence [43]. Moreover, emerging evidence has indicated that biosynthesis regulation of circRNAs is similar to the counterpart of protein-coding genes with their expression regulated by corresponding promoter methylation status [44]. These findings drove us to ask whether the promoter methylation may be implicated in the revealed expression changes of m^6^A-circHECA in SHFs of cashmere goats during SHF cycle. Therefore, we further investigated the promoter methylation status of m^6^A-circHECA host gene (*HECA*) at three SHF stages of cashmere goats including telogen, anagen and catagen. As observed from Figure 6d, a CpG island of 649-bp was revealed in promoter region directly upstream to the TSS of *HECA* gene. Within the CpG island region, we amplified a fragment with a length of 276-bp that contained 26 CpG sites and spanned multiple potential binding sites of transcription factors, such as AP-2α, Sp1, c-Fos, NF-1, c-Jun, and SRF (Figure 6e).

We performed the detection on methylation status of analyzed *HECA* gene promoter region in nine analyzed DNA samples pooled into three stage groups: telogen, anagen and catagen. As a result, the ratios of methylated CpG sites are 12.31%, 1.92%, and 2.69% at telogen, anagen and catagen, respectively (Figure 6f). Taken together with the express change of m^6^A-circHECA in SHFs of cashmere goats among telogen, anagen and catagen (Figure 6a), the telogen exhibited lower expression of m^6^A-circHECA, but had the higher methylation level in analyzed promoter region of *HECA* gene. In contrast, both anagen and catagen exhibited higher expression of m^6^A-circHECA, but had lower methylation level in analyzed promoter region of *HECA* gene. Thus, it appears that a higher methylation level of promoter region of *HECA* gene in SHFs of cashmere goats may cause the lower expression of m^6^A-circHECA (Figure 6a; 6f). Recently, a highly similar case was also reported in multiple myeloma cells where the decreasing expression of circRNA-ciRS-7 was found to be associated with promoter hypermethylation of its host gene LINC00632 [45]. On the other hand, however, it was also reported that some circRNAs could regulate the promoter methylation of their host genes via other epigentica factors [46]. As an example, the circRNA-FECR1 transcribed from *FLI1* gene was found to recruit TET1 (a demethylase) binding to the promoter of its host gene further to cause the hypomethylation in *cis* [47]. Therefore, we strongly recommend further investigate whether the m^6^A-circHECA in turn regulates the promoter methylation level of its host gene (*HECA*) in SHFs of cashmere goats through forming a feedback loop mechanism.

Meanwhile, although the precise mechanism on how epigenetic machinery regulates the biogenesis of circRNAs in cells is largely unknown, taken together with our results, it can be inferred that the lower promoter methylation of *HECA* gene most likely is implicated in higher expression of m^6^A-circHECA at both anagen and catagen SHFs of cashmere goats, which might explain, at least in part, the changed expression of m^6^A-circHECA in SHFs of cashmere goat among the analyzed three stages: telogen, anagen and catagen (Figure 6a; 6d). These results provided further information from epigenetic layer for understanding the regulatory mechanism on m^6^A-circHECA expression in SHFs of cashmere goats.

## CONCLUSION

The circHECA was verified to contain four m^6^A modification sites in SHFs of cashmere goats including m^6^A-213, m^6^A-297, m^6^A-780, and m^6^A-927, and its expression was detected mainly in cytoplasm of SHF stem cells of cashmere goats. The m^6^A-circHECA may play multiple roles in SHF physiology process of cashmere goats through potentially both miRNA mediated target genes and directly or indirectly interaction with target proteins. The promoter methylation of m^6^A-circHECA host gene (*HECA*) most likely is essentially implicated in its expression inhibition in SHFs of cashmere goats.

## Figures and Tables

**Table 1:** Detailed information of polymerase chain reaction primers used in the present investigation with the corresponding amplicon size and annealing temperature

Gene/site name	Reference	Sequence (5′-3′)^1)^	Primer length (nt)	Amplicon size (bp)	Annealing temperature (°C)
m^6^A-circHECA (Divergent primers)	Yin et al [8]	F:ACGATGTTCCCTGTCACCTT	20	87	57
		R:TCGTCTTTCTCCAGGTCCAC	20		
*circHECA-m6A-213*	This study	F:TGGACCTGGAGAAAGACG	18	132	54
		R:GGCCGATGCAGTTGAACT	18		
*circHECA-m6A-297*	This study	F:CTTCTGCTCCTGCCGCTG	18	96	53
		R:CCCGACTTCTTCTTCTTC	18		
*circHECA-m6A-780*	This study	F:GCGCCGGTGCAGTTCCTC	18	108	56
		R:CATCTGGGCATCGTCCTC	18		
*circHECA-m6A-927*	This study	F:CGAGGACCTGCGCAAGTT	18	100	57
		R:CTGCTCGAACACCGGGAG	18		
*GAPDH*	Yin et al [12]	F:TGAACCACGAGAAGTATAACAACA	24	125	53
		R:GGTCATAAGTCCCTCCACGAT	21		
*snRNA-U6*	Han et al [48]	F: CGCTTCGGCAGCACATATAC	20	na	55
		R:AAATATGGAACGCTTCACGA	20		
*HECA* (BSP-primers)	NC_030816.1 in Genbank^2)^	F:AGGGTTATTTTAGGAAGGTAGG	22	276	57
		R:AAACAAAAAACTCTCCAACC	20		

na, not available.

1) F: forward, R: reverse.

2) GenBank: https://www.ncbi.nlm.nih.gov
